# Optimization of laser capture microdissection and RNA amplification for gene expression profiling of prostate cancer

**DOI:** 10.1186/1471-2199-8-25

**Published:** 2007-03-21

**Authors:** Dagmar M Kube, Cemile D Savci-Heijink, Anne-Françoise Lamblin, Farhad Kosari, George Vasmatzis, John C Cheville, Donald P Connelly, George G Klee

**Affiliations:** 1 Department of Laboratory Medicine and Pathology, Mayo Clinic College of Medicine, 200 First St. S.W., Rochester, Minnesota 55905, USA; 2 Comprehensive Cancer Center, Mayo Clinic College of Medicine, 200 First St. S.W., Rochester, Minnesota 55905, USA; 3 Department of Laboratory Medicine and Pathology, University of Minnesota, 425 Delaware St. S.E., Minneapolis, Minnesota 55455, USA; 4 Cancer Center Informatics Shared Resource, University of Minnesota, 425 Delaware St. S.E., Minneapolis, Minnesota 55455, USA; 5 Fish & Richardson P.C., 60 South Sixth Street, Minneapolis, Minnesota 55402, USA

## Abstract

**Background:**

To discover prostate cancer biomarkers, we profiled gene expression in benign and malignant cells laser capture microdissected (LCM) from prostate tissues and metastatic prostatic adenocarcinomas. Here we present methods developed, optimized, and validated to obtain high quality gene expression data.

**Results:**

RNase inhibitor was included in solutions used to stain frozen tissue sections for LCM, which improved RNA quality significantly. Quantitative PCR assays, requiring minimal amounts of LCM RNA, were developed to determine RNA quality and concentration. SuperScript II™ reverse transcriptase was replaced with SuperScript III™, and SpeedVac concentration was eliminated to optimize linear amplification. The GeneChip^® ^IVT labeling kit was used rather than the Enzo BioArray™ HighYield™ RNA transcript labeling kit since side-by-side comparisons indicated high-end signal saturation with the latter. We obtained 72 μg of labeled complementary RNA on average after linear amplification of about 2 ng of total RNA.

**Conclusion:**

Unsupervised clustering placed 5/5 normal and 2/2 benign prostatic hyperplasia cases in one group, 5/7 Gleason pattern 3 cases in another group, and the remaining 2/7 pattern 3 cases in a third group with 8/8 Gleason pattern 5 cases and 3/3 metastatic prostatic adenocarcinomas. Differential expression of alpha-methylacyl coenzyme A racemase (AMACR) and hepsin was confirmed using quantitative PCR.

## Background

Gene expression was profiled in prostate cells to discover candidate biomarkers for early detection of prostate cancer and assessment of cancer aggressiveness. Specific populations of benign and malignant cells were collected from frozen prostate tissues using laser capture microdissection (LCM). RNA was isolated from the cells and amplified to obtain sufficient quantities of labeled antisense RNA for microarray expression profiling. Numerous publications have described amplification methods [1-15] and linear amplification [16] has been used extensively. Optimization and standardization of methods will likely improve overall correlations between microarray studies [17], especially for experiments involving LCM. Methods upstream of RNA amplification, including frozen tissue processing, are crucial for preserving RNA integrity and obtaining accurate results from microarray experiments. A complete protocol for LCM, linear amplification of RNA, and microarray expression profiling is presented here. The protocol includes a quantitative PCR (qPCR) method for determining RNA concentration and integrity that is amenable to limited quantities of RNA obtained from LCM samples. Confirmation of microarray results using qPCR is also presented.

## Results

### Optimization of RNA quality in tissues processed for LCM

**Figure 1:**
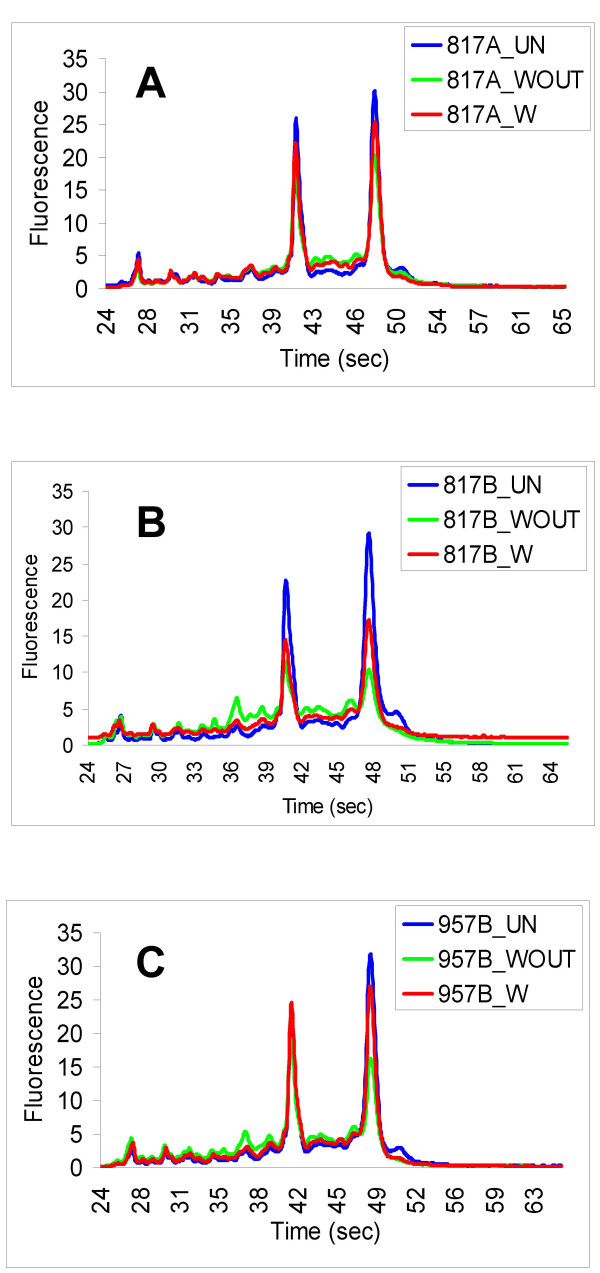
**Preservation of RNA integrity by inclusion of RNase inhibitor in solutions for staining frozen tissue sections**. (A-C), electropherograms of total RNA samples from serial sections of frozen tissues that were unstained (UN), stained in the absence of RNase inhibitor (WOUT), or stained in the presence of RNase inhibitor (W).

The quality of total RNA extracted from stained tissues was assessed using an Agilent bioanalyzer [18]. Significant RNA degradation occurred during the staining protocol, probably due to reactivation of endogenous nucleases in aqueous solutions. RNase inhibitor was, therefore, included in all staining solutions except xylene, in which it is insoluble. Electropherograms of total RNA extracted from serial sections of three representative prostate tissue specimens that were unstained, stained in the absence of RNase inhibitor, or stained in the presence of RNase inhibitor are presented in Fig. 1. Relative to unstained tissues, RNA degradation occurred in tissues stained in the absence of RNase inhibitor, as evidenced by a shortening and broadening of the 28S ribosomal RNA peak at about 48 seconds. In all cases, degradation was decreased when tissues were stained in the presence of RNase inhibitor, as evidenced by recovery of the 28S ribosomal RNA peak height.

Quantitative measures of RNA degradation, Degradation Factors and RNA Integrity Numbers [18,19], based on the electropherograms presented in Fig. 1 are provided [see Additional file 1]. These quantitative measures were in accordance with visual interpretations. We also developed a qPCR assay to characterize RNA integrity using primer pairs specific for the 5' and 3' ends of the PSA transcript. The qPCR results [see Additional file 2] correlated well with quality assessments based on electrophoretic traces. Interestingly, tissues that appeared similar in terms of RNA integrity when unstained were observed to differ significantly in RNA degradation after staining, especially in the absence of RNase inhibitor.

### LCM and determination of RNA concentration and quality

Specific cell populations based on primary Gleason pattern [20,21] were collected using LCM. Epithelial cells were captured from five benign tissues and two cases with benign prostatic hyperplasia (BPH). Primary Gleason pattern 3, 4, and 5 cells were collected from seven, two, and eight cases, respectively. Tumor cells were also collected from three cases of prostate cancer lymph node metastases. LCM images taken before, during, and after capture of primary Gleason pattern 3 cells are presented [see Additional file 3].

We attempted to use a NanoDrop^® ^spectrophotometer to quantify the limited amounts of LCM RNA because the small sample requirement (1–2 μL) precludes the need for sample dilution. Measurements typically were not reproducible, presumably because the sample concentrations were at or below the detection limit of 1.5 ng/μL [22]. Determination of RNA quality using the Agilent bioanalyzer also lacked reproducibility, even though the total RNA Pico assay has a qualitative range of 200–5000 pg/μL [23]. We, therefore, developed a qPCR assay to assess RNA quantity and integrity.

A primer set specific for the 3' end of the β-actin transcript was designed to quantify relative RNA concentrations. β-actin was used for normalization rather than GAPDH because GAPDH expression levels have been shown to correlate with pathologic stage in human prostate tumors [24]. The 3' β-actin primer set was combined with another primer set specific for the middle (M) region of the β-actin transcript to determine 3'/M β-actin ratios for assessment of RNA quality. The primer sets are designated 3' and M because the amplicons they generate fall within the target sequences used to design the 3' and M beta-actin probes on the Affymetrix U133 Plus 2.0 array. The ratio of the RNA quantities determined using the two primer sets is indicative of RNA quality. This is because oligo(dT) primer is used to synthesize cDNA template for amplification in qPCR, allowing only transcripts with intact 3' ends to be detected. Transcripts shortened due to degradation are detected as having less amplification of their upstream ends and, therefore, higher 3'/M ratios.

**Figure 2:**
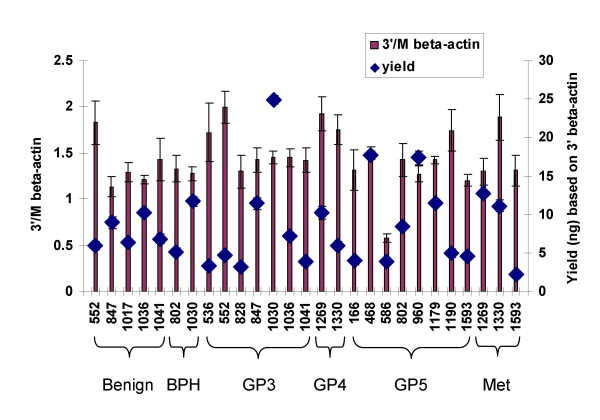
**Yield and integrity of RNA isolated from laser capture microdissected cells**. (Left axis), 3'/M beta-actin ratios determined by qPCR as a measure of the quality of RNA samples obtained by LCM of a total of 27 cases of benign prostate, benign prostatic hyperplasia (BPH), primary Gleason pattern 3 (GP3), pattern 4 (GP4), pattern 5 (GP5), and metastatic prostate cancer (Met). (Right axis), relative yields of LCM RNA samples based on qPCR with primers specific for the 3' end of the beta-actin transcript.

Results of the analysis of RNA quality and concentration based on qPCR with β-actin primers are presented in Fig. 2 for samples obtained after LCM of 27 prostate tissues. Since the 3'/M β-actin ratios were all two or lower, this was established as a quality control parameter for proceeding with linear amplification. One case, 586-GP5, was observed to be an outlier with a 3'/M beta-actin ratio of 0.6, and this result was reproduced. Yields of RNA obtained for the different cases varied substantially. Yield was not observed to correlate with quality since even samples with low yield (e.g., 1593-GP5) had good quality RNA. We, therefore, sought to determine if there was a correlation between yield and number of cells collected for the different cases. We used the number of laser pulses performed during capture of cells as an approximation of the number of cells collected for each case. There was no discernable correlation between yield and number of laser pulses or tissue type [see Additional file 4]. Individual cases appear to differ significantly in RNA content, making it difficult to determine how many cells must be collected to obtain sufficient RNA for linear amplification.

### Comparison of the IVT and Enzo kits

To determine whether to use the IVT kit [25,26] or the Enzo kit in our linear amplification protocol, we performed a side-by-side comparison. Samples of total RNA from Gleason pattern 3 and Gleason pattern 5 microdissected cells were amplified and labeled using each kit. Different amounts of total RNA from Gleason pattern 3 and Gleason pattern 5 cells, 0.7 ng and 5.3 ng, respectively, were amplified to determine if 2- to 10-fold less than the minimum recommended amount of input RNA could yield sufficient labeled cRNA for hybridization to a microarray. This was important since it was not feasible to obtain 10–100 ng of RNA from microdissected cells for most of the 27 prostate tissues analyzed (Fig. 2). Furthermore, to avoid concentrating the RNA [see Additional file 5], the total amount of RNA needed for linear amplification should be contained in 4 μL or less (see Materials and Methods). This volume is limited by the minimum volume of buffer, 11 μL, needed to elute RNA from the purification column.

**Figure 3:**
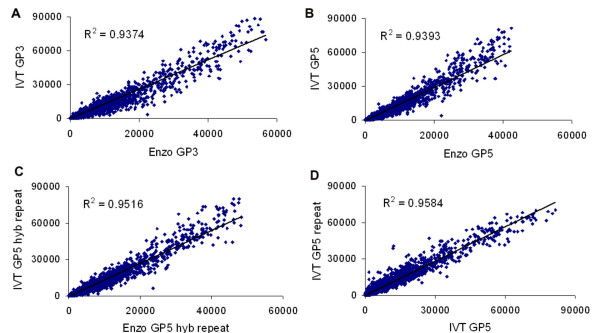
**Signal correlation scatter plots for inter-assay and intra-assay comparisons of the IVT and Enzo transcript labeling kits**. Samples of total RNA from Gleason pattern 3 (GP3) and pattern 5 (GP5) laser capture microdissected cells were linearly amplified, labeled using the IVT kit or the Enzo kit, and hybridized to HG-U133 Plus 2.0 arrays. Signal correlation of inter-assay analysis comparing GP3 samples (A), GP5 samples (B), and hybridization replicates (see Results) of GP5 samples (C) labeled using the IVT or Enzo kit. (D), signal correlation of intra-assay analysis comparing replicate GP5 samples amplified on different days starting with 2 ng or 5 ng of total RNA, labeled using the IVT kit, and scanned using two different GeneChip^® ^Scanner 3000 instruments. Note that the intensity level ranges for transcripts labeled with the IVT and Enzo kits are different.

A sufficient yield of labeled cRNA (21–72 μg) was obtained for each sample to hybridize 15 μg to a U133 Plus 2.0 array. Correlation plots were generated using un-normalized expression data from GCOS 1.1. Signal correlations (R^2^) were 0.94 for inter-assay comparisons (Figures 3A and 3B). Saturation of signal intensities for transcripts expressed at higher levels was apparent for targets prepared using the Enzo kit since the plots assumed a banana shape at higher signals. In agreement with these results, previous studies indicated that use of the Enzo kit to prepare targets for hybridization to the U133 Plus 2.0 array resulted in some degree of saturation of signal intensities for transcripts expressed at very high levels compared with data obtained on the U133A array, and the IVT kit alleviates the high-end saturation effect observed with the Enzo kit [25]. We chose to use the IVT kit in our linear amplification protocol.

We investigated the reproducibility of the final steps of the procedure, namely fragmentation of additional aliquots of the Gleason pattern 5 cRNA samples labeled using the IVT kit or the Enzo kit, hybridization of the fragmented cRNA to U133 Plus 2.0 arrays from a different lot, washing, staining, and scanning (referred to as hybridization replicates). Enzo saturation was again evident in the correlation plot of the hybridization replicates, and the inter-assay signal correlation (R^2^) was 0.95 (Fig. 3C). For comparison, Fig. 3D shows a scatter plot correlation of expression data from replicate Gleason pattern 5 samples amplified and labeled on different days using the IVT kit. The replicates were treated the same except that different amounts of total RNA (approximately 2 ng and 5 ng) were used for amplification, and the microarrays were scanned using two different GeneChip^® ^Scanner 3000 instruments. The intra-assay correlation (R^2 ^= 0.96) was better than the inter-assay correlations (R^2 ^= 0.94–0.95), and no banana shape was evident.

The hybridization replicates allowed another interesting analysis to be performed. Signal correlation (R^2^) between replicate hybridizations was plotted against signal intensity for Gleason pattern 5 samples labeled using the IVT kit or the Enzo kit. Correlations between hybridization replicates decreased rapidly with decreasing signal intensity both for samples labeled using the IVT kit and the Enzo kit [see Additional file 6]. Average signal intensities obtained with the IVT kit were significantly lower than those obtained with the Enzo kit, shifting the entire plot to the left. Signal correlations between hybridization replicates represented a best-case scenario; therefore, replicates of the entire linear amplification procedure were also analyzed. For any given intensity value, the correlation was significantly higher between the hybridization replicates than the replicates of the entire linear amplification protocol.

### Linear amplification of LCM RNA

Equivalent amounts (about 2 ng) of total RNA from normal epithelial cells, BPH cells, Gleason pattern 3 cells, Gleason pattern 5 cells, and lymph node metastases that were microdissected from 25 total cases were amplified and labeled using the IVT kit as described in Methods. SuperScript™ III, rather than SuperScript™ II, was used to synthesize cDNA since it was found to be more processive [see Additional file 7]. The average yield of labeled cRNA was 72 μg (ranging from 42–94 μg). The average size of the biotinylated cRNA was about 750 base pairs for each of the 25 cases. The average fold amplification during the first round was 60-fold, which was significantly greater than the 4–10-fold recommended by the Working Group [27]. The average fold amplification during the second round was about 800-fold, whereas it should have been about 400-fold according to the Working Group [27]. A so-called "normalizer sample" was amplified along with the other 25 samples. This sample consisted of RNA from microdissected Gleason pattern 4 cells. Aliquots of the normalizer sample were stored at -80°C to allow one aliquot to be amplified with each subsequent batch of test samples to serve as a replicate and an indicator of batch-to-batch variability.

**Figure 4:**
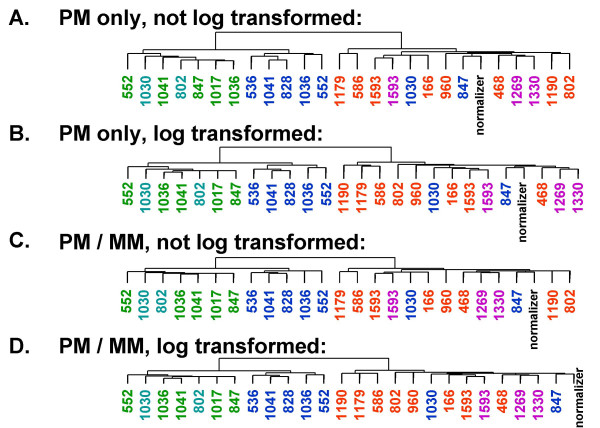
**Unsupervised clustering based on gene expression profiling of benign prostatic epithelial, primary prostate cancer, and metastatic prostate cancer cells laser capture microdissected from 26 cases**. Results of unsupervised clustering using the dChip PM-only model (A and B) or the PM/MM difference model (C and D) to calculate expression values, which were (B and D) or were not (A and C) log transformed. Approximately 1500–2000 genes with the highest standard deviation/mean (not log transformed) or the highest standard deviation (log transformed) were used in the analysis. Green: benign; teal: benign prostatic hyperplasia; blue: Gleason pattern 3; orange: Gleason pattern 5; pink: metastatic prostate cancer; normalizer: normalizer sample (Gleason pattern 4, see Results).

Probesets detected as present were binned into low, medium, and high expression level categories [see Additional file 8] [see Additional file 9]. The coefficient of variance for the number of probesets in each bin was generally low (less than 6%). We observed no apparent correlation between the yield or RNA integrity based on the 3'/M β-actin assay and the number of probesets in different bins. Unsupervised clustering using less than 2000 genes grouped 5/5 normal and 2/2 BPH cases in one clade, 5/7 Gleason pattern 3 cases in another clade, and the remaining 2/7 pattern 3 cases in a third clade that also included 8/8 Gleason pattern 5 cases along with 3/3 lymph node metastases (Fig. 4). The normalizer sample (Gleason pattern 4) clustered with the high-grade samples.

Principal components analysis [see Additional file 10] showed segregation of samples along Eigengene vector 1 to be predominately by Gleason pattern. From left to right, a progression was observed from benign prostatic epithelial and BPH cells to Gleason pattern 3, Gleason pattern 5, and metastatic cells.

**Figure 5:**
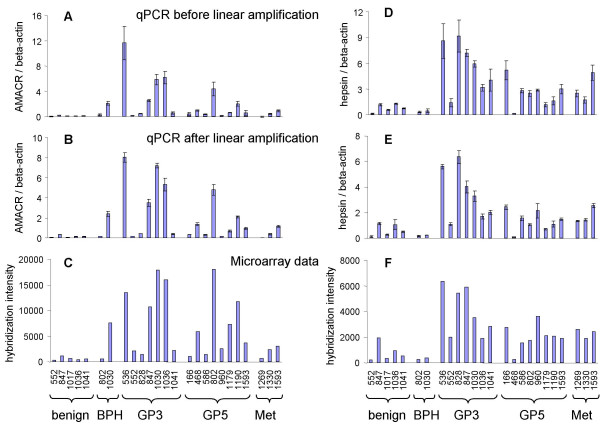
**Confirmation of microarray data using qPCR**. Relative expression levels of AMACR (A-C) and hepsin (D-F) in benign prostatic epithelial, primary prostate cancer, and metastatic prostate cancer cells laser capture microdissected from 25 cases were measured using qPCR (A, B, D, E) and HG-U133 Plus 2.0 arrays (C and F). Quantitative PCR was performed using cDNA synthesized from RNA samples before (A and D) and after (B and E) linear amplification. For microarray analysis, RNA samples were linearly amplified and labeled using the IVT kit.

We used qPCR to confirm differential expression of alpha-methylacyl coenzyme A racemase (AMACR) and hepsin detected using microarrays. Aliquots of the same samples that were amplified and labeled to generate microarray results were also analyzed by qPCR before and after linear amplification. Relative expression levels of AMACR and hepsin measured by qPCR in unamplified samples correlated well with results obtained with amplified samples and with microarray results (Fig. 5). Both AMACR and hepsin were upregulated in prostate cancer compared to benign prostatic epithelial cells. The mean fold difference for AMACR expression between prostate cancer and benign cells was 5.3 (p = 0.02) in unamplified samples, 4.5 (p = 0.014) after linear amplification, and 4.2 (p = 0.004) as determined by microarray analysis. The mean fold difference for hepsin expression between prostate cancer and benign cells was 5.7 (p = 0.00004) for unamplified samples, 4.4 (p = 0.0003) after linear amplification, and 4.3 (p = 0.00003) as determined by microarray analysis. The fidelity of differential gene expression was, therefore, preserved during the linear amplification procedure. Correlations between microarray and qPCR data after linear amplification were 0.83 and 0.87 for AMACR and hepsin expression, respectively. Expression of hepsin was highest on average in Gleason pattern 3 cells (5.6 ± 2.9 hepsin/beta-actin ratio), and was observed to decrease in high grade, Gleason pattern 5 and lymph node metastasis, samples (2.6 ± 1.5) to a level intermediate between benign (0.7 ± 0.4) and low grade, Gleason pattern 3, cells (Figures 5D–5F). Interestingly, a similar pattern was observed for AMACR expression (Figures 5A–5C).

## Discussion and conclusion

Specific cell populations were captured based on primary Gleason pattern, and their gene expression profiles were analyzed. In general, unsupervised clustering grouped cases in accordance with Gleason pattern. However, the grouping was not perfect in this regard since two of seven Gleason pattern 3 cases clustered with Gleason pattern 5 cases and lymph node metastases. The molecular profile may provide more detailed information than the Gleason pattern, indicating that the Gleason 3 cases are more aggressive, or have the potential to be more aggressive, than expected based on histology alone.

In agreement with previous results [28-35] we found that the type II transmembrane serine protease hepsin is approximately 5-fold overexpressed in prostate cancer cells compared to benign prostatic epithelial cells. Furthermore, metastatic cells and Gleason pattern 5 cells, which grouped together in unsupervised clustering, had hepsin expression levels intermediate between Gleason pattern 3 and benign cells. Hepsin immunohistochemistry has indicated that the staining intensity of hormone-refractory metastatic cancers is intermediate between localized prostate tumors (Gleason score 6–8) and benign prostate [29]. The lymph node metastasis cases we analyzed were not hormone-refractory, indicating that hepsin expression may not be regulated by hormones. Also in agreement with previously results [29,31,35-37] we observed up-regulation of AMACR expression in prostate cancer relative to benign cells (about 5-fold). The expression pattern of AMACR was similar to that of hepsin in that AMACR expression in high grade, Gleason pattern 5 and metastatic cancer, cells was intermediate between low grade, Gleason pattern 3, and benign cells. Interestingly, AMACR expression has been reported to be lower in hormone-refractory metastatic prostate cancer than hormone-naïve-localized prostate cancer [37], suggesting that AMACR protein expression might be regulated by androgens. A subsequent report [38] suggests, however, that AMACR expression is not hormone-dependent, but that it may be a marker of tumor differentiation. This is consistent with the data presented here showing decreased AMACR expression in Gleason pattern 5 cells and non-hormone-refractory lymph node metastases relative to Gleason pattern 3 cells. Interestingly, in a recently published gene expression study of laser microbeam microdissected populations of prostate cancer, prostatic intraepithelial neoplasia (PIN), and normal prostatic epithelial cells [39] hepsin was not found to be differentially expressed between normal epithelium and PINs and prostate tumors, but AMACR was found to be up-regulated.

Recommendations of the Working Group [27] for fold amplification during the first and second rounds of linear amplification may not be based on experiments where minimum amounts of input RNA were amplified (less than 200 ng). Therefore, the relevance of these recommendations to such experiments is unclear. The ability to successfully amplify small amounts of total RNA is becoming increasingly important. LCM is labor-intensive, especially given the need for significant numbers of biological replicates when analyzing human specimens. Minimizing the requirements for input RNA can have a significant impact on study feasibility. In addition, interest in analyzing individual cells continues to grow [40].

Standards for the size of biotinylated cRNA obtained from two cycle amplification protocols are needed. For one cycle experiments, the Working Group recommends that the biotinylated cRNA should be 500–3,000 base pairs in size, and that samples that do not meet these criteria should be discarded [27]. However, two cycle amplification protocols are biased to the 3' ends of transcripts.

We developed qPCR assays to assess RNA quality and quantity. Our qPCR assay for RNA quality is analogous to the 3'/5' and 3'/M ratios for GAPDH and β-actin that are quality control parameters for Affymetrix microarrays [41]. Arcturus has also provided a qPCR protocol to assess RNA quality in formalin fixed paraffin embedded tissues [42]. Quantitative PCR assays are fairly reproducible [see Additional file 11] and extremely sensitive, requiring significantly less sample than the NanoDrop^® ^ND-1000 spectrophotometer and the Agilent 2100 bioanalyzer for analysis of RNA quality and quantity. In addition, qPCR is a more direct, functional assay for mRNA quality than analysis of ribosomal RNA as a surrogate for mRNA using the Agilent bioanalyzer. It is also noteworthy that measurements of absorbance at 260 nm can be misleading because degraded RNA will also contribute to the absorbance, which could give an erroneously high estimate of intact RNA concentration.

Using the U133 Plus 2.0 arrays, the reliability of the signal was observed to decrease dramatically with decreased intensity [see Additional file 6]. It may be possible to increase the sensitivity by hybridizing larger amounts of labeled targets to the arrays. As expected, correlations between hybridization replicates were significantly better than correlations between replicates of the entire protocol performed on different days. The correlation between biological replicates would be expected to be even less.

We compared the IVT and ENZO kits using signal correlation plots. However, it is difficult to determine which kit is superior in the absence of a comprehensive set of known differences in gene expression. A better comparison could be achieved by performing spike-in experiments [25,26] or comprehensive qPCR confirmation of differentially expressed genes.

## Methods

### Frozen tissue sectioning

Surgical specimens with written patient consent were obtained from the Mayo Clinic Specialized Program of Research Excellence in Prostate Cancer (SPORE) tumor bank with Institutional Review Board approval (#1937-00). After radical prostatectomy, tissues from patients that had not received preoperative hormonal therapy, chemotherapy, or radiation therapy were flash frozen in liquid nitrogen and stored at -80°C. Fisherbrand Superfrost uncharged slides (Fisher Scientific, Hampton, NH) were baked at 220°C for at least two hours. Frozen tissue sections were cut at 5 μm in a -20°C cryostat and placed on slides pre-chilled at 4°C. After the tissue adhered to the slide, the slide was immediately placed on a flat piece of dry ice. The slides were placed in a chilled slide box on dry ice, transported under dry ice, and stored at -80°C. Sections from each tissue specimen were stained with hematoxylin and eosin and analyzed by a pathologist.

### Staining of frozen tissue sections, LCM, and RNA isolation

Frozen tissue sections were stained using the HistoGene™ LCM Frozen Section Staining Kit (Arcturus, Mountain View, CA) according to the manufacturer's protocol [43], with the following modifications. ProtectRNA™ RNase inhibitor (1× final concentration, catalog number R7397, Sigma-Aldrich) was added to each solution, except xylene. Slides were transferred on dry ice from the -80°C freezer to the laboratory bench and immediately placed in 75% ethanol. Incubation in 100% ethanol was extended to five minutes. Following incubation in xylene for five minutes, slides were dried for 60 seconds under vacuum in a dessicator.

To analyze RNA integrity in stained tissue sections, RNA was prepared by pipetting 100 μL of extraction buffer directly onto the tissue section on the glass slide and using the pipet tip to gently scrape the tissue into the buffer, which was then transferred into an RNase-free microcentrifuge tube. RNA was extracted and purified using the PicoPure™ RNA Isolation Kit (Arcturus) according to the manufacturer's protocol, including on-column DNase treatment (Qiagen, Valencia, CA).

Immediately after staining a tissue section, LCM was performed by a pathologist using a PixCell^® ^II instrument (Arcturus). Thirty minutes on average (± 15 minutes) were spent collecting cells from a tissue section. RNA was extracted from the captured cells and purified as described above. Extracts from homogeneous populations of cells captured on multiple caps from one to six serial sections of each case were combined onto a single RNA purification column to achieve a sufficient yield and concentration of RNA for linear amplification.

RNA quality was assessed using an Agilent 2100 bioanalyzer (Agilent Technologies, Palo Alto, CA) and/or a qPCR assay for 3'/5' PSA or 3'/M β-actin (described below). RNA quantity was assessed using a NanoDrop^® ^ND-1000 spectrophotometer (NanoDrop Technologies, Wilmington, DE) and/or a qPCR assay for the 3' end of the beta-actin transcript (described below).

### Quantitative PCR

First-strand cDNA synthesis was performed using SuperScript™ II or III (Invitrogen, Carlsbad, CA) and oligo(dT)_12–18 _(Invitrogen) according to the manufacturer's instructions. One μL of LCM RNA was used per 20 μL cDNA synthesis reaction. Typically, 0.25 μL of the resulting cDNA reaction was used per qPCR reaction, and samples were analyzed in triplicate. Primer sequences are provided [see Additional file 12]. Quantitative PCR was performed using a 7900HT instrument (Applied Biosystems, Foster City, CA) and SYBR^® ^Green PCR master mix (Applied Biosystems). Thermal cycle parameters were as follows: 50°C for 2 minutes, 95°C for 10 minutes, 40 cycles of denaturation at 95°C for 15 seconds and annealing and extension at 64°C for one minute. Dissociation curves were generated by denaturation at 95°C for 15 seconds and annealing at 60°C for 15 seconds followed by a gradual increase in temperature (2% ramp rate) to 95°C. Standard curves were generated for each primer pair with serial dilutions of cDNA prepared using a mixture of total RNA isolated from benign and malignant prostate tissues. The results from the tested samples were compared to the corresponding standard curves generated in the same experiment to determine the sample RNA yield. Melting curve analysis was used to assess PCR specificity.

### Target synthesis and hybridization

Target amplification and labeling were performed according to the Affymetrix protocol [41], with the following modifications. Since the starting amount of total RNA was only two ng, the poly-A RNA control stock was diluted five-fold more than recommended for a starting amount of 10 ng of total RNA. The T7-oligo(dT) primer/poly-A controls mix was prepared two-fold more concentrated than recommended to allow one μL to be combined with four μL of total RNA sample for first cycle, first-strand cDNA synthesis. The total RNA sample/T7-oligo(dT) primer/poly-A controls mix was incubated at 65°C for three minutes. SuperScript™ III (Invitrogen) was used to synthesize cDNA for one hour at 50°C. In the second cycle, purified cRNA was incubated with random primers for five minutes at 65°C. SuperScript™ III (Invitrogen) was used to synthesize first-strand cDNA for one hour at 50°C. First-strand cDNA was incubated with T7-oligo(dT) primer for three minutes at 65°C in preparation for second-strand cDNA synthesis. After cleanup of double-stranded cDNA, 0.5 μL was saved for qPCR analysis, and the remaining cDNA was used to synthesize biotin-labeled cRNA. Labeling of cRNA transcripts with biotin was performed using the GeneChip^® ^IVT Labeling Kit (IVT kit; Affymetrix, Santa Clara, CA) or the Enzo BioArray™ HighYield™ RNA Transcript Labeling Kit (Enzo kit; Enzo Life Sciences, Farmingdale, NY). Labeling reactions were incubated for 16 hours. Yield of biotinylated cRNA was measured using a NanoDrop^® ^ND-1000 spectrophotometer (NanoDrop Technologies). Unfragmented and fragmented cRNA samples were analyzed on an Agilent 2100 bioanalyzer (Agilent Technologies). Fifteen μg of fragmented cRNA were hybridized to U133 Plus 2.0 arrays (Affymetrix), washed, stained, and scanned according to the Affymetrix protocol [41].

### Data analysis

To generate signal correlation scatter plots for replicate samples prepared with the IVT kit or the Enzo kit, un-normalized expression data from GCOS 1.1 were plotted for all genes except the Affymetrix control genes.

Unsupervised clustering was performed using dChip version 1.3 software. All 26 arrays were first normalized to the array with median intensity (1041-GP3). The PM-only or PM/MM model was used with expression values that were or were not log_2 _transformed. Approximately 1500–2000 genes with the highest standard deviation/mean (not log_2 _transformed) or the highest standard deviation (log_2 _transformed) were used to perform unsupervised hierarchical clustering.

## Abbreviations

LCM – laser capture microdissected

AMACR – alpha-methylacyl coenzyme A racemase

qPCR – quantitative PCR

GAPDH – Glyseraldehyde-3-phosphate dehydrogenase

BPH – benign prostatic hyperplasia

## Authors' contributions

DMK was involved in designing, coordinating, and carrying out the experiments described in this report, and she wrote the manuscript. CDS-H was involved in designing and performing the Laser Capture Microdissection. A-FL was involved in the review of data and editing of manuscript. GV supervised the design and implementation of the overall process. FK was involved in QC data analysis, producing the data in response to the review, and editing of the manuscript. JCC supervised all issues related to pathology and design of the experiments. DPC was involved in overall design of the study and review of the data. GGK was involved in the overall design of study, review of data, and editing of manuscript. All authors read and approved the final manuscript.

## Supplementary Material

Additional File 1 Quantitative measures of RNA degradation, DegFact and RIN, based on electrophoretic traces shown in Fig. 1. Larger DegFact (scale of 0–100%) and smaller RIN (scale of 1–10) indicate more degradation. 817A: prostate cancer tissue; 817B: matched benign prostate tissue; 957B: benign prostate tissue; UN: unstained; WOUT: stained without RNase inhibitor; W: stained in the presence of RNase inhibitor; YELLOW: degradation can be detected; ORANGE: severe degradation; RED: highest alert, strong degradation. To standardize interpretation of RNA integrity, quantitative measures of RNA degradation based on electropherograms have been developed. With increasing degradation, heights of 18S and 28S peaks gradually decrease and additional 'degradation peak signals' appear in a molecular weight range between small RNAs and the 18S peak [19]. The degradation factor (DegFact, %Dgr/18S) is defined as the ratio of the average degradation peak signal (30–41 seconds) to the 18S peak signal (41–42.5 seconds) multiplied by 100 [19]. The larger the degradation factor, the more degraded the sample. The RNA Integrity Number (RIN) allows for classification of eukaryotic total RNA based on a numbering system from 1 to 10, with 1 being the most degraded and 10 being the most intact [18]. Degradation factors and RINs based on the electropherograms in Figures 1A–1C are listed in Additional file 1. For all three cases, degradation factors were higher and RINs were lower for sections stained in the absence of the RNase inhibitor relative to serial sections that were unstained. Degradation factors were decreased and RINs were increased for tissues stained in the presence compared to the absence of RNase inhibitor, indicating a protective effect against RNA degradation. Thus, the quantitative measures were in accordance with visual interpretations. Interestingly, tissues that appeared similar in terms of RNA integrity when unstained were observed to differ significantly in RNA degradation after staining, especially in the absence of RNase inhibitor. As shown in Additional file 1, the degradation factors were similar for 817A (9.7) and 817B (9.6) when the tissues were unstained; however, the degradation factors for 817A (14.6) and 817B (30.9) were very different after staining without RNase inhibitor. Even in the presence of RNase inhibitor, a significant difference remained between 817A (12.6) and 817B (18.5). Thus, differences in tissue quality that are not apparent before staining can become evident after staining. It can be more informative, therefore, to analyze tissue sections for RNA integrity after fixing, staining, and dehydrating rather than analyzing unstained tissues. Click here for file

Additional File 2 3'/5' PSA ratios determined by qPCR as a measure of RNA integrity to analyze the effect of preparing sections on cold or room temperature slides and staining in the presence or absence of RNase inhibitor. To characterize RNA integrity, we developed a qPCR assay using primer sets specific for the 5' and 3' ends of the PSA transcript. The ratio of the RNA quantity determined using the two primer sets is indicative of RNA quality in the sample. Larger 3'/5' ratios indicate greater degrees of RNA degradation. This is because oligo(dT) primer is used to synthesize cDNA template for amplification in qPCR, allowing only transcripts with intact 3' ends to be detected. Transcripts shortened due to degradation are detected as having less amplification of their 5' ends and, therefore, higher 3'/5' ratios. Additional file 2 shows 3'/5' ratios of PSA generated by qPCR of cDNA prepared using RNA from the same samples analyzed in Figures 1A–1C. The qPCR results correlate well with quality assessments based on electrophoretic traces. By any measure, 817B stained without RNase inhibitor was observed to be the most degraded followed by 817B stained in the presence of RNase inhibitor. Additional file 2 also shows a consistent trend towards higher 3'/5' PSA ratios for frozen sections placed on glass slides at room temperature compared to serial sections prepared on cold (4°C) slides prior to quick freezing on dry ice and storing at -80°C. 817A: prostate cancer tissue; 817B: matched benign prostate tissue; 957B: benign prostate tissue. Click here for file

Additional File 3 LCM of Gleason pattern 3 cells using the AutoPix™ instrument. (A) before capture, (B) selection of cells for capture (highlighted in red), (C) procurement of cells by binding to the cap membrane, (D) after capture, and (E) captured cells (HistoGene™ stain, ×100). Click here for file

Additional File 4 Number of laser pulses performed during LCM does not correlate with RNA yield. This graph plots the number of laser pulses versus the yield of total RNA for cells collected from the indicated tissue types using the indicated laser spot sizes. BPH: benign prostatic hyperplasia; GP3: Gleason pattern 3; GP5: Gleason pattern 5; met: metastatic prostate cancer. Click here for file

Additional File 5 SpeedVac concentration degrades RNA. The Affymetrix protocol for two-cycle cDNA synthesis [41] calls for 10–100 ng of total RNA in a volume of 3 μL or less, necessitating a minimum concentration of about 3 ng/μL. This was not obtained for any of the 27 cases. The effect of SpeedVac concentration on RNA integrity was therefore investigated. A 20 μL sample of LCM RNA was concentrated to 1 μL in a SpeedVac, followed by addition of 19 μL of nuclease-free water. This sample was analyzed side-by-side with an equivalent amount of the same sample prior to concentration using an Agilent 2100 bioanalyzer. The 28S ribosomal RNA peak for the concentrated sample was significantly shorter than that of the sample that was not concentrated, indicating that the RNA was degraded during concentration (upper graph). Linear amplification of RNA degraded by SpeedVac concentration resulted in a significantly smaller size distribution of labeled cRNA (lower graph). Therefore, LCM RNA samples were not concentrated. Click here for file

Additional File 6 Signal correlation between replicates plotted against signal intensity. Samples of total RNA from Gleason pattern 5 (GP5) laser capture microdissected cells were linearly amplified, labeled using the IVT or Enzo kit, and hybridized to HG-U133 Plus 2.0 arrays. Hybridization replicates and replication of the entire procedure using the IVT kit were performed as described in Results. Signal correlation between replicate samples was plotted against signal intensity using expression data generated with dChip version 1.3. Replicates prepared using the IVT kit were normalized relative to each other, and replicates prepared using the Enzo kit were normalized as a separate group since average signal intensities were significantly lower with the IVT kit than the Enzo kit. The PM-only model was used to calculate expression values, which were not log transformed. Probe sets, excluding Affymetrix controls, were then sorted by expression level for one of the replicate samples in each group. Signal correlations between replicates were calculated for the top 20,000 expression values divided into 20 consecutive bins, each with 1000 expression values. The maximum signal intensity in each bin was plotted against the correlation between replicate signals across that bin. The minimum signal in each bin is approximately equal to the maximum signal in the next (lower signal intensity) bin, which is plotted in the graph. Click here for file

Additional File 7 SuperScript™ III is more processive than SuperScript™ II. The Affymetrix protocol for eukaryotic target preparation [41] calls for use of SuperScript™ II reverse transcriptase to synthesize cDNA. According to Invitrogen, SuperScript™ III outperforms SuperScript™ II in terms of producing high yields and full-length cDNA [46]. We, therefore, compared the two enzymes. One μL of each of six different RNA samples generated by LCM of prostate cancer tissue was used in a cDNA synthesis reaction with SuperScript™ II or SuperScript™ III. Equivalent amounts of the cDNA reactions were used in qPCR reactions with primer pairs specific for the 3' or 5' ends of the PSA transcript. Quantitative PCR with primers specific for the 3' end of PSA showed consistently higher transcript levels in the cDNA samples synthesized using SuperScript™ II compared to those synthesized with SuperScript™ III (top graph). However, primers specific for the 5' end of PSA detected equivalent amounts of transcripts in samples synthesized by the two different enzymes (middle graph). Thus, lower 3'/5' PSA ratios were consistently achieved for cDNA samples synthesized using SuperScript™ III compared to samples synthesized using SuperScript™ II (bottom graph). These results indicate that SuperScript™ III is more processive than SuperScript™ II because it generates longer transcripts rather than generating a larger number of shorter transcripts. This is important because oligonucleotide probes on Affymetrix arrays are selected within regions 600 nucleotides upstream of transcript ends [47]. We, therefore, used SuperScript™ III for linear amplification of RNA. Click here for file

Additional File 8 Number of undetected (Absent) probesets or Present probesets classified into Low (Intensity < 5.98), Medium (5.98 < Intensity < 7.62), or High (Intensity > 7.62) bins, 3'/M ratios for β-actin, and the total RNA yields based on the 3' β-actin qPCR assay. The intensity thresholds for bins were selected by the analysis of log_2 _transformed expression levels of publicly available U133PLUS2 microarray data on the prostate benign and tumor tissues. [44,45]. The 33 and 66 percentile of the intensity values for the "present" probesets of all the samples in the study were selected for thresholds. Of note, the distribution of probesets in Low, Medium, and High bins is fairly uniform with a coefficient of variance < 6% for the three bins. Click here for file

Additional File 9 Number of undetected (Absent) probesets or Present probesets classified into Low (Intensity < 6.15), Medium (6.15 < Intensity < 8.17), or High (Intensity > 8.17) bins. Thresholds for the Low, Medium, and High intensity bins were selected by identifying the 5 percentile (about 4.1) and 95 percentile (about 10.2) intensity values for the probesets in Varambally et al. data. [44,45] and dividing the range into three equal bins. Column designations are as described [see Additional file 8]. Click here for file

Additional File 10 PCA of Loess normalized microarray data. Genedata Expressionist (Genedata, Basel, Switzerland) was used to perform principal components analysis following normalization of gene expression values using the LOWESS algorithm. Samples tend to segregate along the primary Eigengene vector from left to right according to increasing level of pathologic state ranging from benign to metastatic cases. Click here for file

Additional File 11 Reproducibility plot of qPCR assay. Measurements were repeated on different days for 8 cDNA samples using primers specific for 3' and 5' regions of PSA. The agreement between the two measurements is generally very good. Click here for file

Additional File 12 Primers used for quantitative PCR. The sequences of the primers used for the qPCR assays are provided. Click here for file
