# Supplementary material for: Optimization of laser capture microdissection and RNA amplification for gene expression profiling of prostate cancer
Source: BMC Mol Biol. 2007 Mar 21;8:25. doi: 10.1186/1471-2199-8-25 (PMC1847526; doi:10.1186/1471-2199-8-25)
Supplement: Additional File 1 — Quantitative measures of RNA degradation, DegFact and RIN, based on electrophoretic traces shown in Fig. 1. Larger DegFact (scale of 0–100%) and smaller RIN (scale of 1–10) indicate more degradation. 817A: prostate cancer tissue; 817B: matched benign prostate tissue; 957B: benign prostate tissue; UN: unstained; WOUT: stained without RNase inhibitor; W: stained in the presence of RNase inhibitor; YELLOW: degradation can be detected; ORANGE: severe degradation; RED: highest alert, strong degradation. To standardize interpretation of RNA integrity, quantitative measures of RNA degradation based on electropherograms have been developed. With increasing degradation, heights of 18S and 28S peaks gradually decrease and additional 'degradation peak signals' appear in a molecular weight range between small RNAs and the 18S peak [19]. The degradation factor (DegFact, %Dgr/18S) is defined as the ratio of the average degradation peak signal (30–41 seconds) to the 18S peak signal (41–42.5 seconds) multiplied by 100 [19]. The larger the degradation factor, the more degraded the sample. The RNA Integrity Number (RIN) allows for classification of eukaryotic total RNA based on a numbering system from 1 to 10, with 1 being the most degraded and 10 being the most intact [18]. Degradation factors and RINs based on the electropherograms in Figures 1A–1C are listed in Additional file 1. For all three cases, degradation factors were higher and RINs were lower for sections stained in the absence of the RNase inhibitor relative to serial sections that were unstained. Degradation factors were decreased and RINs were increased for tissues stained in the presence compared to the absence of RNase inhibitor, indicating a protective effect against RNA degradation. Thus, the quantitative measures were in accordance with visual interpretations. Interestingly, tissues that appeared similar in terms of RNA integrity when unstained were observed to differ significantly in RNA degradation after stainin [file 1471-2199-8-25-S1.ppt]

## Slide 1
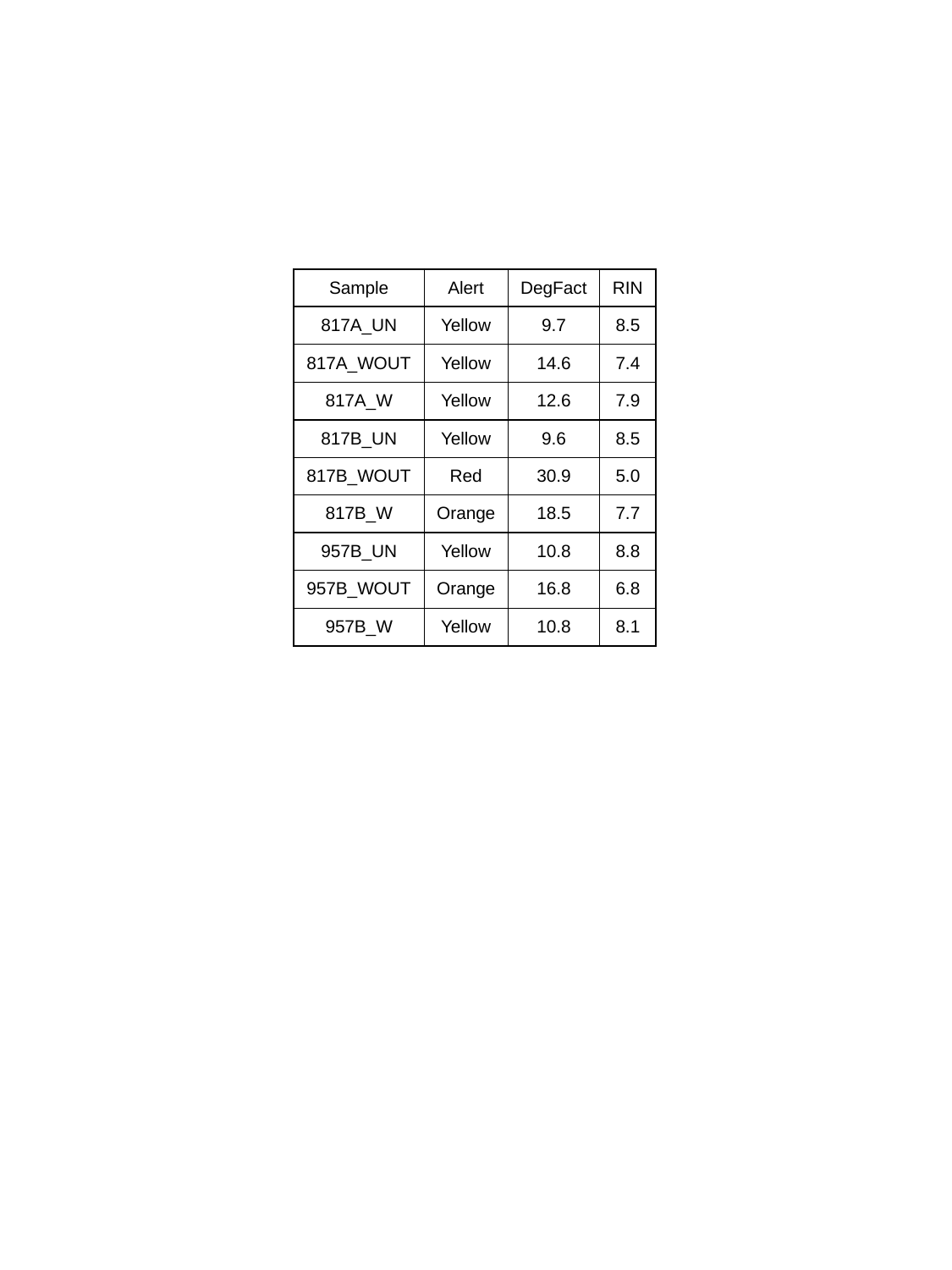

| Sample | Alert | DegFact | RIN |
| --- | --- | --- | --- |
| 817A\_UN | Yellow | 9.7 | 8.5 |
| 817A\_WOUT | Yellow | 14.6 | 7.4 |
| 817A\_W | Yellow | 12.6 | 7.9 |
| 817B\_UN | Yellow | 9.6 | 8.5 |
| 817B\_WOUT | Red | 30.9 | 5.0 |
| 817B\_W | Orange | 18.5 | 7.7 |
| 957B\_UN | Yellow | 10.8 | 8.8 |
| 957B\_WOUT | Orange | 16.8 | 6.8 |
| 957B\_W | Yellow | 10.8 | 8.1 |
