# Supplementary figures and images for: Optimization of laser capture microdissection and RNA amplification for gene expression profiling of prostate cancer
Source: BMC Mol Biol. 2007 Mar 21;8:25. doi: 10.1186/1471-2199-8-25 (PMC1847526; doi:10.1186/1471-2199-8-25)

## Slide 1
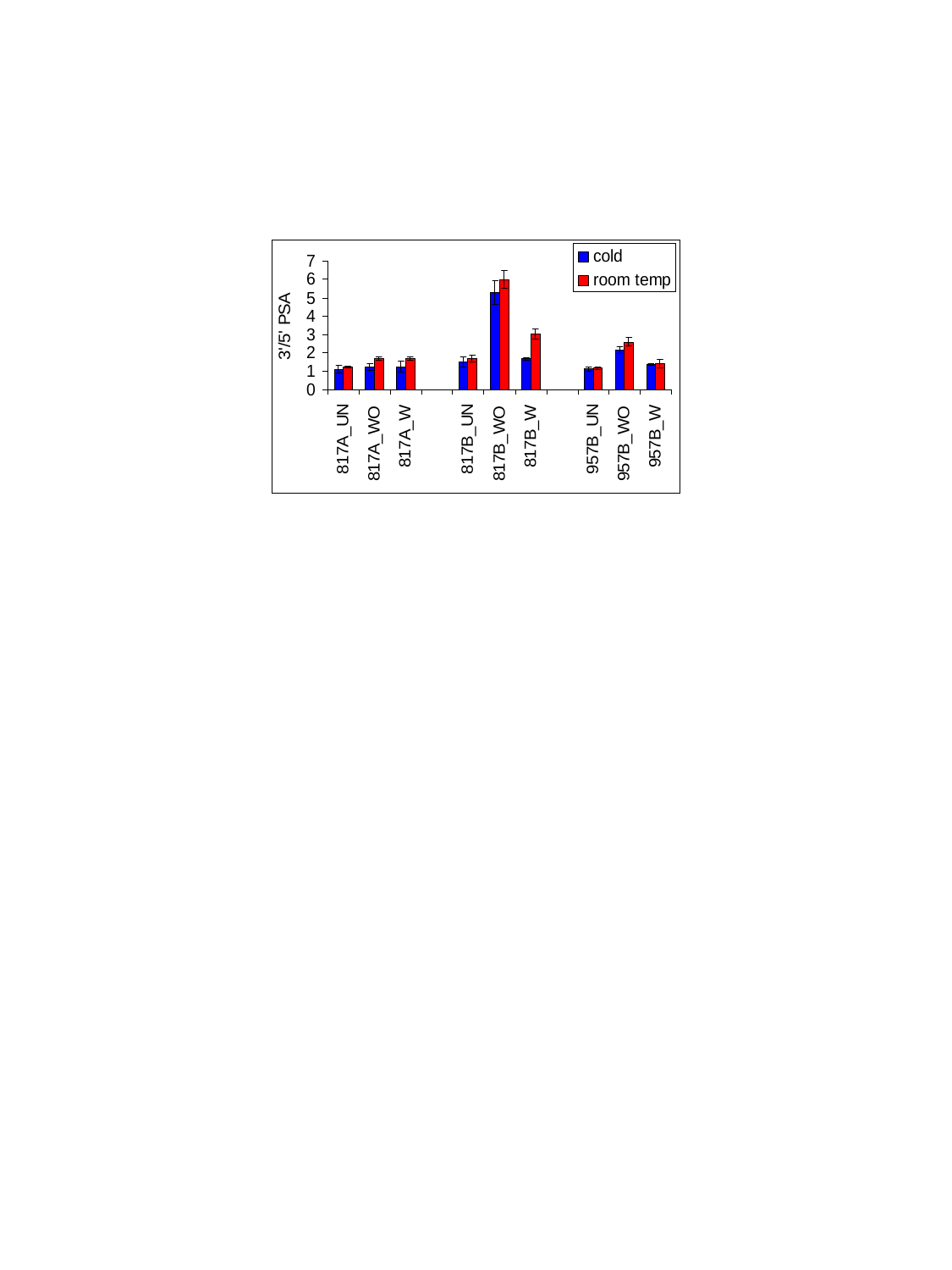

Supplement: Additional File 2 — 3'/5' PSA ratios determined by qPCR as a measure of RNA integrity to analyze the effect of preparing sections on cold or room temperature slides and staining in the presence or absence of RNase inhibitor. To characterize RNA integrity, we developed a qPCR assay using primer sets specific for the 5' and 3' ends of the PSA transcript. The ratio of the RNA quantity determined using the two primer sets is indicative of RNA quality in the sample. Larger 3'/5' ratios indicate greater degrees of RNA degradation. This is because oligo(dT) primer is used to synthesize cDNA template for amplification in qPCR, allowing only transcripts with intact 3' ends to be detected. Transcripts shortened due to degradation are detected as having less amplification of their 5' ends and, therefore, higher 3'/5' ratios. Additional file 2 shows 3'/5' ratios of PSA generated by qPCR of cDNA prepared using RNA from the same samples analyzed in Figures 1A–1C. The qPCR results correlate well with quality assessments based on electrophoretic traces. By any measure, 817B stained without RNase inhibitor was observed to be the most degraded followed by 817B stained in the presence of RNase inhibitor. Additional file 2 also shows a consistent trend towards higher 3'/5' PSA ratios for frozen sections placed on glass slides at room temperature compared to serial sections prepared on cold (4°C) slides prior to quick freezing on dry ice and storing at -80°C. 817A: prostate cancer tissue; 817B: matched benign prostate tissue; 957B: benign prostate tissue. [file 1471-2199-8-25-S2.ppt]

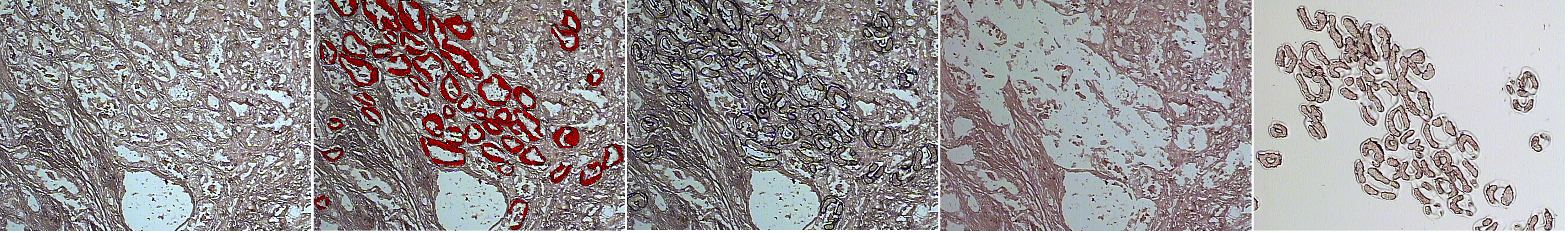

Supplement: Additional File 3 — LCM of Gleason pattern 3 cells using the AutoPix™ instrument. (A) before capture, (B) selection of cells for capture (highlighted in red), (C) procurement of cells by binding to the cap membrane, (D) after capture, and (E) captured cells (HistoGene™ stain, ×100). [file 1471-2199-8-25-S3.jpeg]

## Slide 1
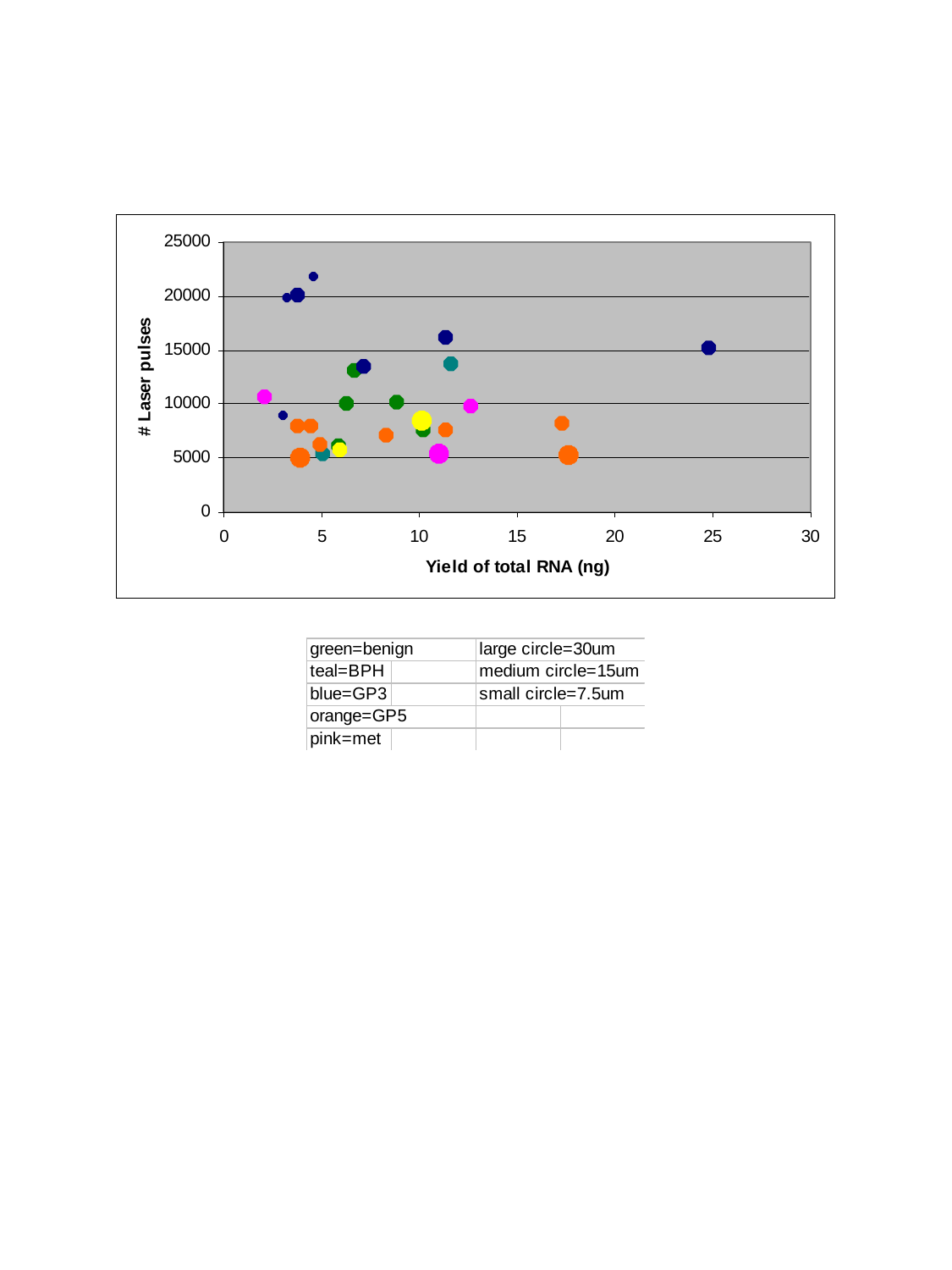

Supplement: Additional File 4 — Number of laser pulses performed during LCM does not correlate with RNA yield. This graph plots the number of laser pulses versus the yield of total RNA for cells collected from the indicated tissue types using the indicated laser spot sizes. BPH: benign prostatic hyperplasia; GP3: Gleason pattern 3; GP5: Gleason pattern 5; met: metastatic prostate cancer. [file 1471-2199-8-25-S4.ppt]

## Slide 1
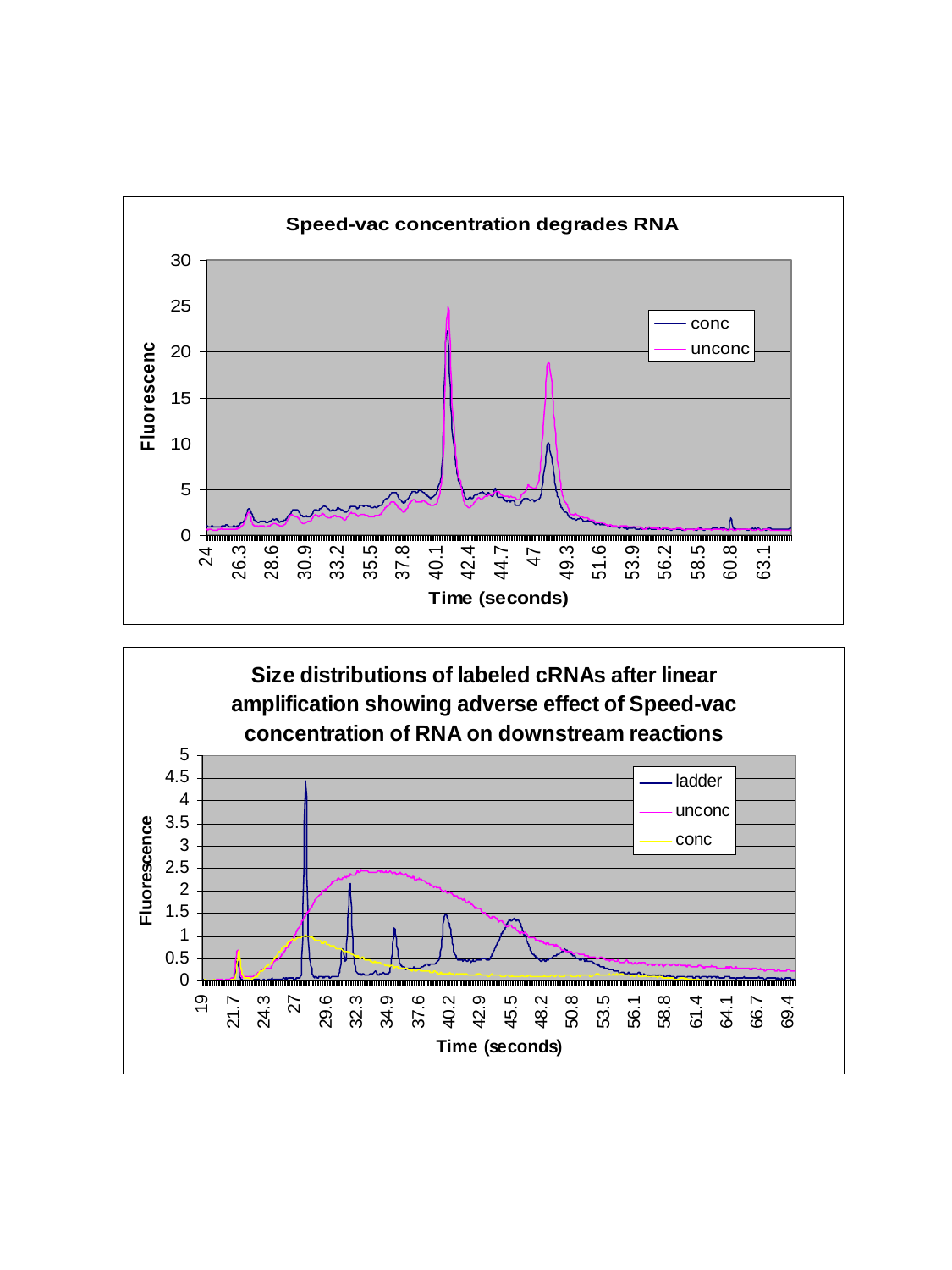

Supplement: Additional File 5 — SpeedVac concentration degrades RNA. The Affymetrix protocol for two-cycle cDNA synthesis [41] calls for 10–100 ng of total RNA in a volume of 3 μL or less, necessitating a minimum concentration of about 3 ng/μL. This was not obtained for any of the 27 cases. The effect of SpeedVac concentration on RNA integrity was therefore investigated. A 20 μL sample of LCM RNA was concentrated to 1 μL in a SpeedVac, followed by addition of 19 μL of nuclease-free water. This sample was analyzed side-by-side with an equivalent amount of the same sample prior to concentration using an Agilent 2100 bioanalyzer. The 28S ribosomal RNA peak for the concentrated sample was significantly shorter than that of the sample that was not concentrated, indicating that the RNA was degraded during concentration (upper graph). Linear amplification of RNA degraded by SpeedVac concentration resulted in a significantly smaller size distribution of labeled cRNA (lower graph). Therefore, LCM RNA samples were not concentrated. [file 1471-2199-8-25-S5.ppt]

## Slide 1
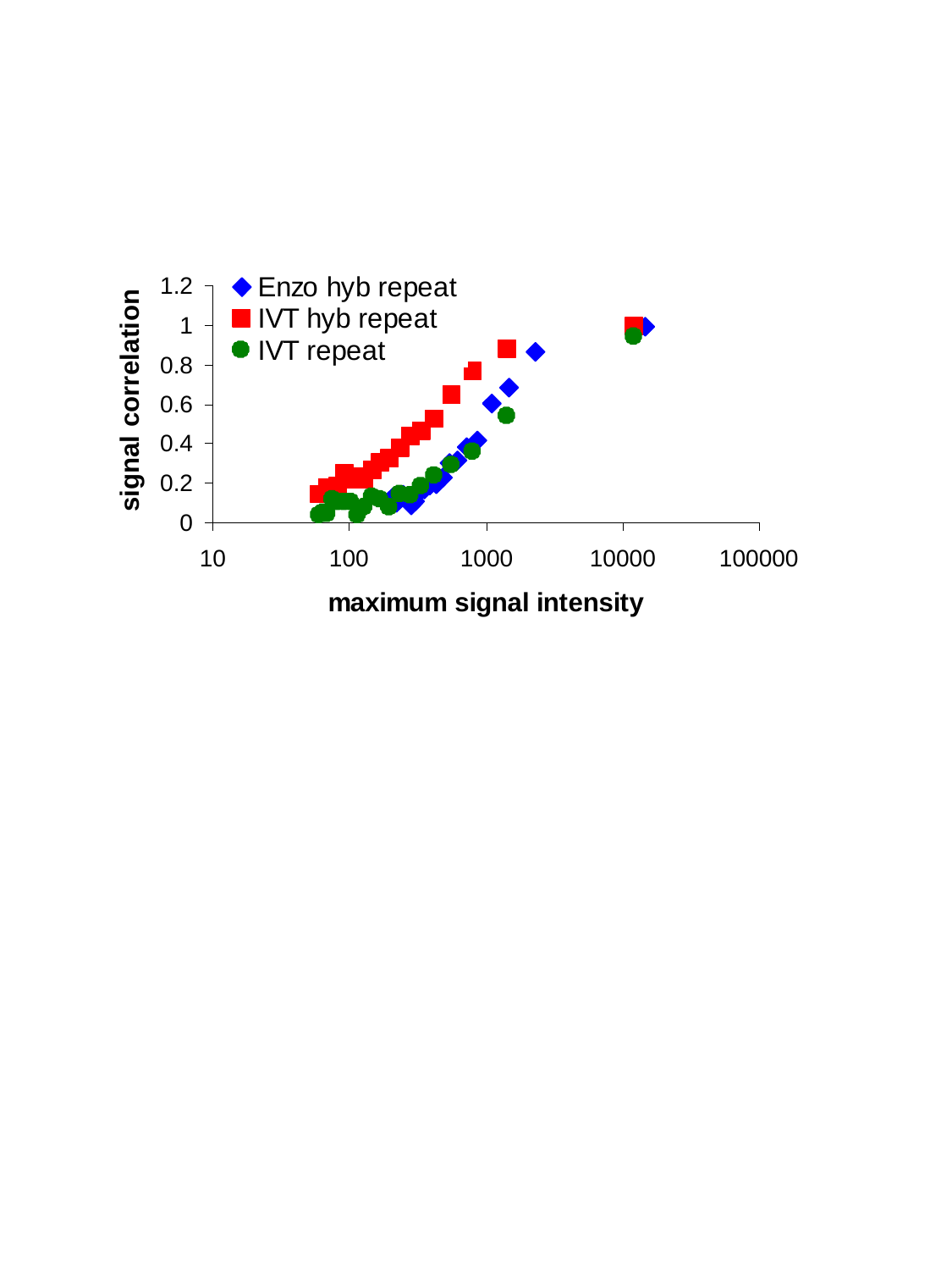

Supplement: Additional File 6 — Signal correlation between replicates plotted against signal intensity. Samples of total RNA from Gleason pattern 5 (GP5) laser capture microdissected cells were linearly amplified, labeled using the IVT or Enzo kit, and hybridized to HG-U133 Plus 2.0 arrays. Hybridization replicates and replication of the entire procedure using the IVT kit were performed as described in Results. Signal correlation between replicate samples was plotted against signal intensity using expression data generated with dChip version 1.3. Replicates prepared using the IVT kit were normalized relative to each other, and replicates prepared using the Enzo kit were normalized as a separate group since average signal intensities were significantly lower with the IVT kit than the Enzo kit. The PM-only model was used to calculate expression values, which were not log transformed. Probe sets, excluding Affymetrix controls, were then sorted by expression level for one of the replicate samples in each group. Signal correlations between replicates were calculated for the top 20,000 expression values divided into 20 consecutive bins, each with 1000 expression values. The maximum signal intensity in each bin was plotted against the correlation between replicate signals across that bin. The minimum signal in each bin is approximately equal to the maximum signal in the next (lower signal intensity) bin, which is plotted in the graph. [file 1471-2199-8-25-S6.ppt]

## Slide 1
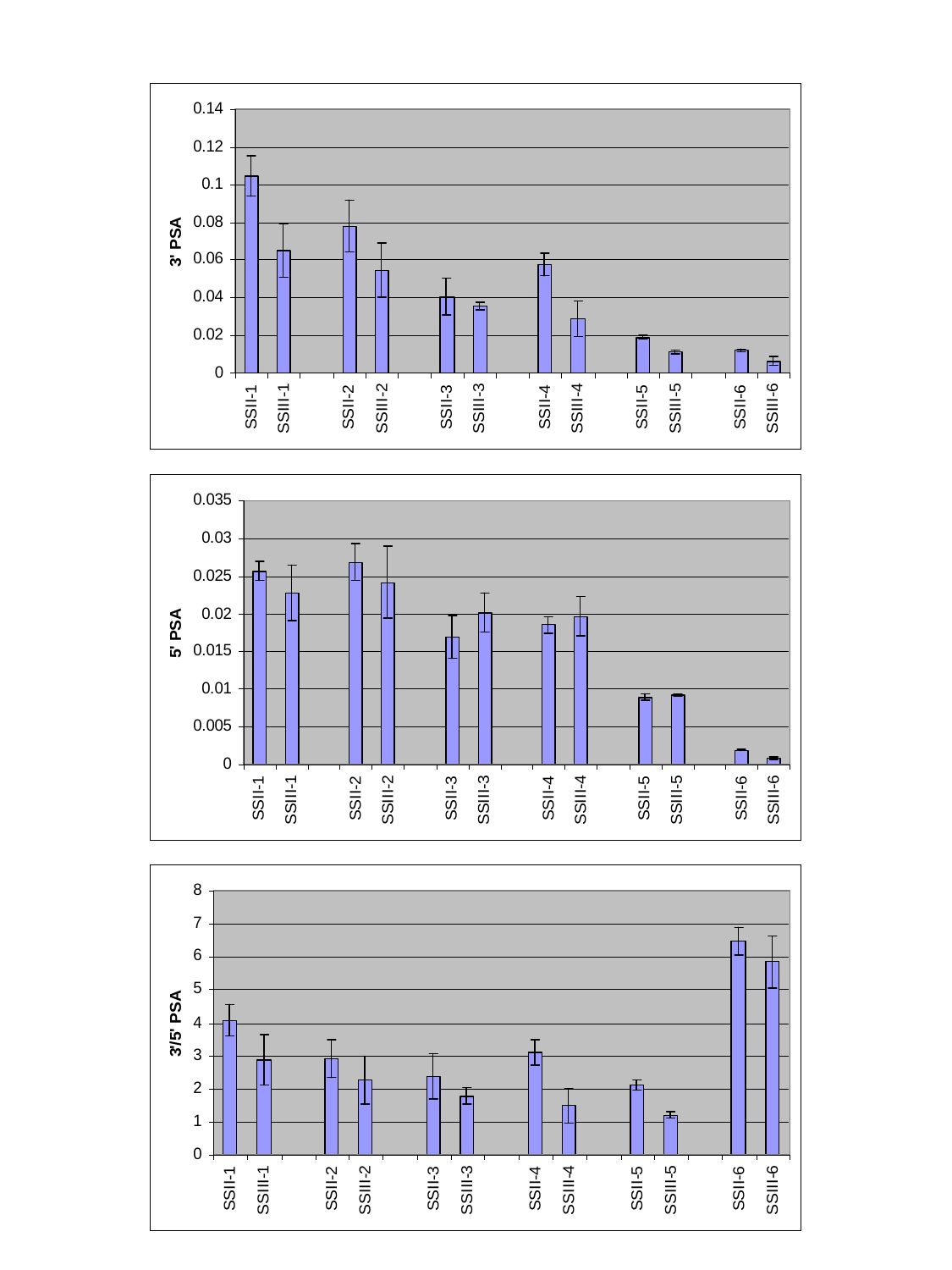

Supplement: Additional File 7 — SuperScript™ III is more processive than SuperScript™ II. The Affymetrix protocol for eukaryotic target preparation [41] calls for use of SuperScript™ II reverse transcriptase to synthesize cDNA. According to Invitrogen, SuperScript™ III outperforms SuperScript™ II in terms of producing high yields and full-length cDNA [46]. We, therefore, compared the two enzymes. One μL of each of six different RNA samples generated by LCM of prostate cancer tissue was used in a cDNA synthesis reaction with SuperScript™ II or SuperScript™ III. Equivalent amounts of the cDNA reactions were used in qPCR reactions with primer pairs specific for the 3' or 5' ends of the PSA transcript. Quantitative PCR with primers specific for the 3' end of PSA showed consistently higher transcript levels in the cDNA samples synthesized using SuperScript™ II compared to those synthesized with SuperScript™ III (top graph). However, primers specific for the 5' end of PSA detected equivalent amounts of transcripts in samples synthesized by the two different enzymes (middle graph). Thus, lower 3'/5' PSA ratios were consistently achieved for cDNA samples synthesized using SuperScript™ III compared to samples synthesized using SuperScript™ II (bottom graph). These results indicate that SuperScript™ III is more processive than SuperScript™ II because it generates longer transcripts rather than generating a larger number of shorter transcripts. This is important because oligonucleotide probes on Affymetrix arrays are selected within regions 600 nucleotides upstream of transcript ends [47]. We, therefore, used SuperScript™ III for linear amplification of RNA. [file 1471-2199-8-25-S7.ppt]

## Slide 1
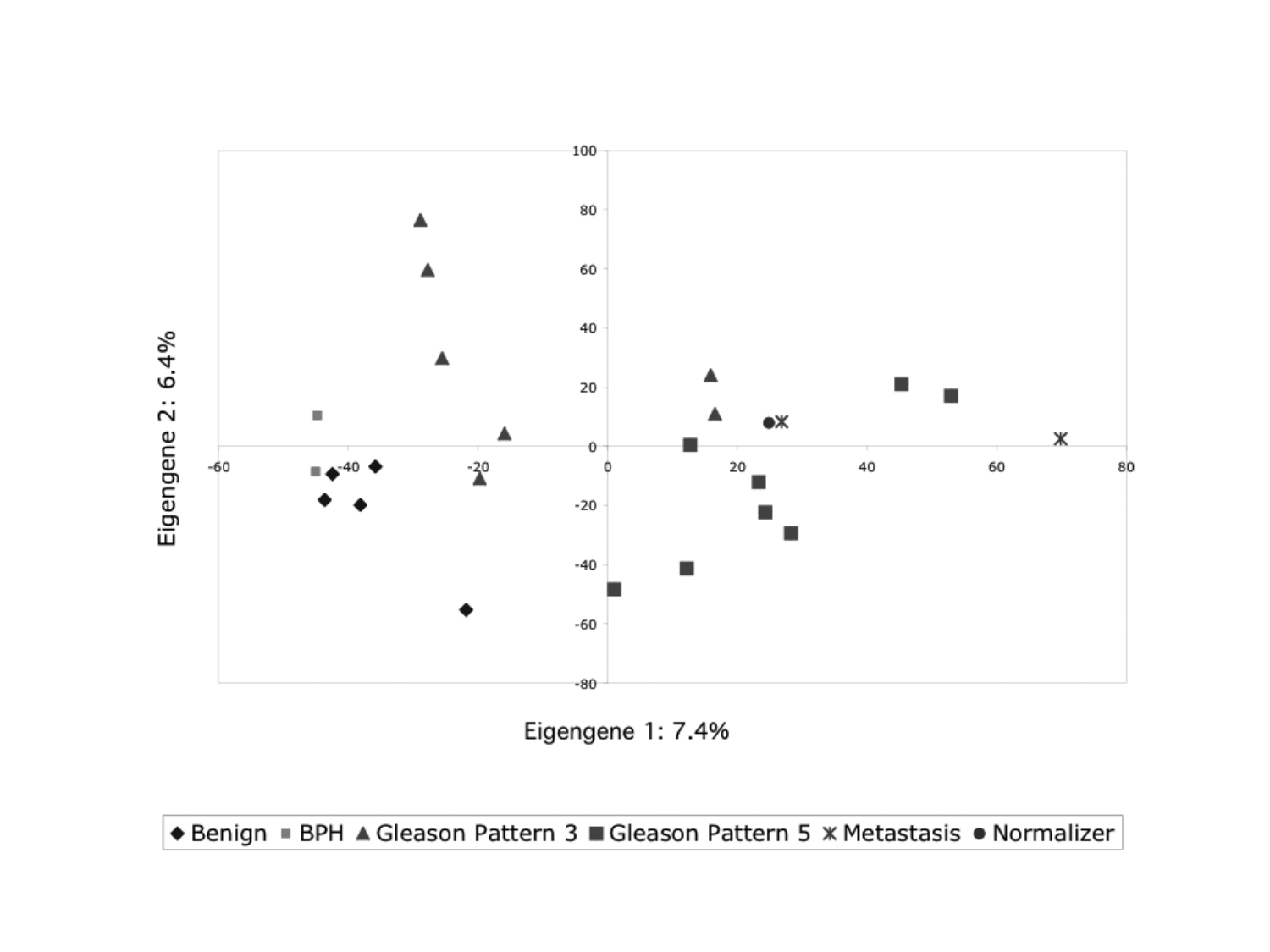

Supplement: Additional File 10 — PCA of Loess normalized microarray data. Genedata Expressionist (Genedata, Basel, Switzerland) was used to perform principal components analysis following normalization of gene expression values using the LOWESS algorithm. Samples tend to segregate along the primary Eigengene vector from left to right according to increasing level of pathologic state ranging from benign to metastatic cases. [file 1471-2199-8-25-S10.ppt]

## Slide 1
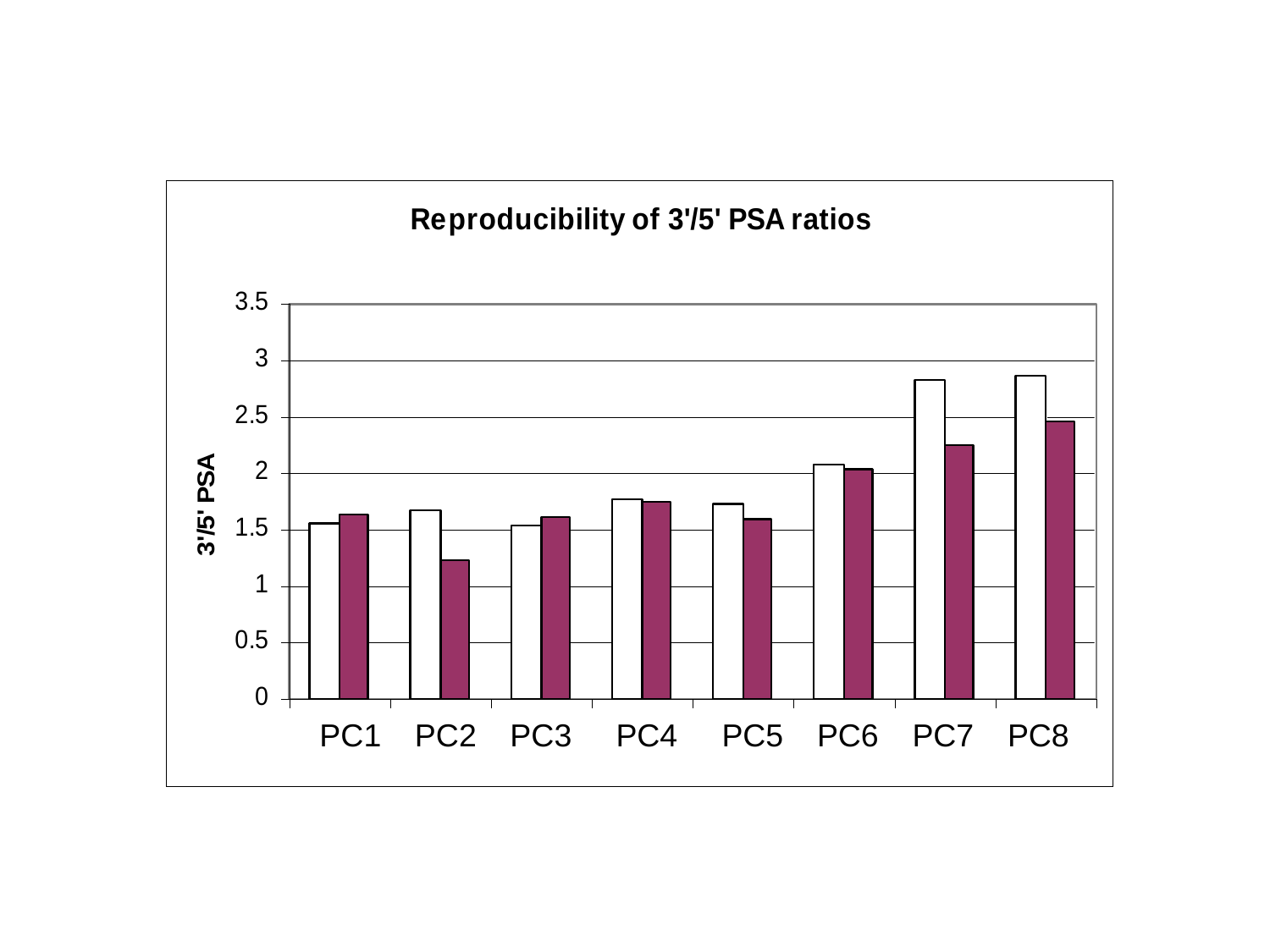

PC1
PC2
PC3
PC4
PC5
PC6
PC7
PC8

Supplement: Additional File 11 — Reproducibility plot of qPCR assay. Measurements were repeated on different days for 8 cDNA samples using primers specific for 3' and 5' regions of PSA. The agreement between the two measurements is generally very good. [file 1471-2199-8-25-S11.ppt]
