# Supplementary material for: Optimization of laser capture microdissection and RNA amplification for gene expression profiling of prostate cancer
Source: BMC Mol Biol. 2007 Mar 21;8:25. doi: 10.1186/1471-2199-8-25 (PMC1847526; doi:10.1186/1471-2199-8-25)
Supplement: Additional File 8 — Number of undetected (Absent) probesets or Present probesets classified into Low (Intensity < 5.98), Medium (5.98 < Intensity < 7.62), or High (Intensity > 7.62) bins, 3'/M ratios for β-actin, and the total RNA yields based on the 3' β-actin qPCR assay. The intensity thresholds for bins were selected by the analysis of log2 transformed expression levels of publicly available U133PLUS2 microarray data on the prostate benign and tumor tissues. [44,45]. The 33 and 66 percentile of the intensity values for the "present" probesets of all the samples in the study were selected for thresholds. Of note, the distribution of probesets in Low, Medium, and High bins is fairly uniform with a coefficient of variance < 6% for the three bins. [file 1471-2199-8-25-S8.doc]

| **Sample** | **High**  **(I > 7.62)** | **Medium**  **(5.98 < I < 7.68)** | **Low**  **(I < 5.98)** | **Absent** | **3’/M ratio**  **(β-actin)** | **Yield (ng) (β-actin)** |
| --- | --- | --- | --- | --- | --- | --- |
| **552** | 8532 | 8360 | 13860 | 23924 | 1.8 | 5.9 |
| **847** | 8288 | 8235 | 15175 | 22978 | 1.1 | 8.9 |
| **1017** | 8218 | 8424 | 15304 | 22730 | 1.3 | 6.4 |
| **1036** | 8054 | 8327 | 14381 | 23914 | 1.2 | 10.2 |
| **1041** | 8272 | 8502 | 15938 | 21964 | 1.4 | 6.8 |
| **802** | 8199 | 8323 | 14971 | 23183 | 1.3 | 5.1 |
| **1030** | 8391 | 8007 | 15091 | 23187 | 1.3 | 11.7 |
| **536** | 7846 | 7826 | 15347 | 23657 | 1.7 | 3.3 |
| **552** | 7858 | 7904 | 13858 | 25056 | 2.0 | 4.7 |
| **828** | 7720 | 7784 | 14414 | 24758 | 1.3 | 3.1 |
| **847** | 7831 | 8368 | 15069 | 23408 | 1.4 | 11.4 |
| **1030** | 7985 | 8755 | 15191 | 22745 | 1.5 | 24.9 |
| **1036** | 8346 | 8267 | 12944 | 25119 | 1.5 | 7.2 |
| **1041** | 7981 | 8397 | 14426 | 23872 | 1.4 | 3.8 |
| **1269** | 7747 | 8239 | 16650 | 22040 | 1.9 | 10.2 |
| **1330** | 7605 | 8229 | 16091 | 22751 | 1.8 | 6.0 |
| **166** | 7346 | 7900 | 14287 | 25143 | 1.3 | 4.0 |
| **468** | 7787 | 7981 | 14748 | 24160 | 1.5 | 17.7 |
| **586** | 8326 | 8725 | 16970 | 20655 | 0.6 | 3.9 |
| **802** | 8153 | 8568 | 14240 | 23715 | 1.4 | 8.4 |
| **960** | 8008 | 8393 | 14395 | 23880 | 1.3 | 17.4 |
| **1179** | 8393 | 8487 | 15207 | 22589 | 1.4 | 11.4 |
| **1190** | 7848 | 8684 | 15284 | 22860 | 1.7 | 5.0 |
| **1593** | 7981 | 8790 | 15243 | 22662 | 1.2 | 4.5 |
| **1269** | 7965 | 8700 | 16021 | 21990 | 1.3 | 12.7 |
| **1330** | 7808 | 8221 | 15037 | 23610 | 1.9 | 11.1 |
| **1593** | 7709 | 8531 | 14830 | 23606 | 1.3 | 2.2 |
| **Ave**  **(SD)** | 8007  (283) | 8331  (289) | 14999 (869) | 23339 (1052) | 1.4  (0.3) | 8  (5.3) |
