# Supplementary material for: Optimization of laser capture microdissection and RNA amplification for gene expression profiling of prostate cancer
Source: BMC Mol Biol. 2007 Mar 21;8:25. doi: 10.1186/1471-2199-8-25 (PMC1847526; doi:10.1186/1471-2199-8-25)
Supplement: Additional File 9 — Number of undetected (Absent) probesets or Present probesets classified into Low (Intensity < 6.15), Medium (6.15 < Intensity < 8.17), or High (Intensity > 8.17) bins. Thresholds for the Low, Medium, and High intensity bins were selected by identifying the 5 percentile (about 4.1) and 95 percentile (about 10.2) intensity values for the probesets in Varambally et al. data. [44,45] and dividing the range into three equal bins. Column designations are as described [see Additional file 8]. [file 1471-2199-8-25-S9.doc]

| **Sample** | **High**  **(I > 8.17)** | **Medium**  **(6.15 < I < 8.17)** | **Low**  **(I < 6.15)** | **Absent** |
| --- | --- | --- | --- | --- |
| **552** | 6092 | 9852 | 14808 | 23924 |
| **847** | 5925 | 9725 | 16048 | 22978 |
| **1017** | 5933 | 9726 | 16287 | 22730 |
| **1036** | 5855 | 9698 | 15209 | 23914 |
| **1041** | 5908 | 9928 | 16876 | 21964 |
| **802** | 5860 | 9679 | 15954 | 23183 |
| **1030** | 6107 | 9458 | 15924 | 23187 |
| **536** | 5707 | 9105 | 16207 | 23657 |
| **552** | 5621 | 9289 | 14710 | 25056 |
| **828** | 5490 | 9165 | 15263 | 24758 |
| **847** | 5593 | 9647 | 16028 | 23408 |
| **1030** | 5669 | 10118 | 16144 | 22745 |
| **1036** | 5998 | 9734 | 13825 | 25119 |
| **1041** | 5731 | 9707 | 15366 | 23872 |
| **1269** | 5581 | 9418 | 17637 | 22040 |
| **1330** | 5381 | 9595 | 16949 | 22751 |
| **166** | 5189 | 9197 | 15147 | 25143 |
| **468** | 5446 | 9490 | 15580 | 24160 |
| **586** | 5939 | 10122 | 17960 | 20655 |
| **802** | 5803 | 9946 | 15212 | 23715 |
| **960** | 5761 | 9742 | 15293 | 23880 |
| **1179** | 6027 | 9868 | 16192 | 22589 |
| **1190** | 5590 | 10041 | 16185 | 22860 |
| **1593** | 5640 | 10120 | 16254 | 22662 |
| **1269** | 5631 | 10061 | 16994 | 21990 |
| **1330** | 5439 | 9721 | 15906 | 23610 |
| **1593** | 5491 | 9819 | 15760 | 23606 |
| **Ave**  **(SD)** | 5719  (233) | 9703  (292) | 15915  (893) | 23339  (1052) |
