# Supplementary material for: Optimization of laser capture microdissection and RNA amplification for gene expression profiling of prostate cancer
Source: BMC Mol Biol. 2007 Mar 21;8:25. doi: 10.1186/1471-2199-8-25 (PMC1847526; doi:10.1186/1471-2199-8-25)
Supplement: Additional File 12 — Primers used for quantitative PCR. The sequences of the primers used for the qPCR assays are provided. [file 1471-2199-8-25-S12.doc]

| PSA 3’ left | 5’-gagggagggtcttcctttgg-3’ |
| --- | --- |
| PSA 3’ right | 5’-ggttgtctggaggacttcaataca-3’ |
| PSA 5’ left | 5’-gaagacacaggccaggtatttca-3’ |
| PSA 5’ right | 5’-gtggctggagtcatcacctg-3’ |
| β-actin 3’ left | 5’-tcctctcccaagtccacaca-3’ |
| β-actin 3’ right | 5’-gcacgaaggctcatcattca-3’ |
| β-actin M left | 5’-gatcattgctcctcctgagc-3’ |
| β-actin M right | 5’-agtccgcctagaagcatttg-3’ |
| AMACR left | 5’-cagtaactcggggcctgttt-3’ |
| AMACR right | 5’-tggatgttgctgtgtgttgg-3’ |
| hepsin left | 5’-ggctgtgtggcattgtgagt-3’ |
| hepsin right | 5’-gctggcttcggagtgagtct-3’ |
